# Proteomics and Mass Spectrometry: What Have We Learned About The Heart?

**DOI:** 10.2174/157340310791162631

**Published:** 2010-05

**Authors:** Shaan Chugh, Colin Suen, Anthony Gramolini

**Affiliations:** 1 Department of Physiology, University of Toronto; 2 Heart and Stroke/Richard Lewar Centre of Cardiovascular Excellence

## Abstract

The emergence of new platforms for the discovery of innovative therapeutics has provided a means for diagnosing cardiac disease in its early stages. Taking into consideration the global health burden of cardiac disease, clinicians require innovations in medical diagnostics that can be used for risk stratification. Proteomic based studies offer an avenue for the discovery of proteins that are differentially regulated during disease; such proteins could serve as novel biomarkers of the disease state. For instance, in clinical practice, the abundance of such biomarkers in blood could be correlated with the severity of the disease state. As such, early detection of biomarkers would enable an improvement in patient prognosis. In this review, we outline advancements in various proteomic platforms used to study the disease proteome and their applications to the field of clinical medicine. Specifically, we highlight the contributions of proteomic-based profiling experiments to the analysis of cardiovascular diseases.

## 1. INTRODUCTION

In clinical practice, diagnosis in the earliest stages of disease progression can prevent further complications and improve patient prognosis. To address this issue, new and innovative means of diagnosing various disease states are essential to ensure that patients are diagnosed early, and treated correctly, in a timely manner. Proteomic-based studies may fill this gap, and discoveries made in the laboratory setting could improve the delivery of healthcare to various patient populations. Generally, proteomic platforms involve the analysis of global expression and function of the entire protein complement [1]. Indeed, the discovery and identification of certain proteins in the disease state may be used as a stepping stone for the assessment of such proteins as potential biomarkers of disease.

Cardiac diseases have been studied through numerous proteomic-based studies. For example, these platforms have been used to analyze myocardial ischemia [2-4], dilated cardiomyopathy [5, 6], hypertrophic cardiomyopathy [7], and heart failure [8-10]. In some cases these strategies may even have aided in the discovery of additional biomarkers of these disease states. Here, we outline progress that has been made in this field and will detail innovative techniques that now potentially could be used to even improve patient prognosis and therapies.

## 2. CLINICAL PROTEOMICS AT THE BEDSIDE

Clinically-based proteomics has a large potential for the development of strategies which aim to alleviate risk associated with cardiac disease. Biomarkers that are used in clinical practice are highly useful in that they support medical decision making, by complementing other diagnostic tests, such as the medical history, physical examination, and various other special tests. Theoretically, there are three criteria that, if satisfied, would provide an optimal biomarker of the disease state. First, the potential biomarkers must be easily measurable in a short time period at a cost that is practical. Second, elevation of this protein would offer diagnostic information that was not previously present in the absence of the protein. Third, the information obtained would aid in the medical decision making process performed by the clinician [11]. Fulfillment of such criteria encourages follow-up of such a biomarker in other model systems or patient cohort samples. For instance, cardiac troponin I has been previously shown to fulfill such criteria, and further follow-up in patient cohorts is underway. Recently, this approach was utilized to ascertain whether Cardiac Troponin I (CTN I) or Creatine Kinase-Myoglobin (CK-MB) could be used as short-term or long-term markers of risk associated with cardiac surgery [12]. In this study, a patient cohort of 252 individuals who had undergone cardiac surgery was used to analyze levels of these two proteins in blood. Not only was CTN-I shown to be a strong predictor of mortality, but increases in the levels of this protein also correlated well with increases in mortality. Findings from this study support the utilization of patient cohorts as a means to ease the transition from bench to bedside. 

## 3. PROTEOMICS: THE NEW FRONTIER

One of the major goals of proteomic studies is to identify and quantify the entire protein complement of a sample, whether that is a purified protein complex, a cellular organelle component, or an entire tissue. A particular interest is the analysis of global protein expression patterns during a pathological state. Obviously, near-complete protein coverage is vital for the elucidation of disease pathways and for the identification of novel proteins involved in the progression of disease. Traditionally, the most common technique used to analyze the protein complement involved gel-based separation, a tool that has proven pivotal in studies focused on protein ‘discovery’, yet has limits to its protein coverage. More recently, improved protein coverage has been obtained through gel-free methods. Here, we outline both of these approaches and their contribution to the field of proteomics of the heart.

### Gel-Based Separation

The first set of ‘large scale’ studies were the gene expression studies examining mRNA levels through arrays with the underlying assumption that there was a correlation between mRNA levels and protein function [13]. This assumption, however, does not always hold true.

Two-dimensional polyacrylamide gel electrophoresis (2DE) was developed as protein levels could be studied in the absence of mRNA or gene expression analysis [14]. Gel-based separation is powerful in that it can also provide insight onto differential protein regulation in the disease state. In 2DE, a current is applied to a complex sample in a 2D gel to separate proteins on the basis of charge and mass. This effectively resolves proteins on the basis of isoelectric point (pI) followed by separation by mass [15]. Groups have employed 2DE with due success in the proteomic analysis of biomarkers of cardiomyopathy [4] and in the investigation of mechanisms underlying cardiomyopathy [16-18] and diabetic cardiomyopathy [19].

2DE-based proteomic studies are highly useful in that they are capable of quantifying expression of complex protein samples which can contain up to 1000’s of proteins [20]. Furthermore, this technique is advantageous in that in provides information regarding the presence of isoforms or post-translational modifications in addition to protein expression levels [21]. One of the limits to this approach is the dynamic range of 2DE which is roughly only ~10^4^; accordingly, there is a pressing need to improve this detection capability [22]. Several strategies are employed to address this problem. One is the depletion of highly abundant proteins prior to gel-based separation. A second strategy is to selectively enrich low-abundance proteins using immunoaffinity ligands, while diluting high-abundance proteins, prior to resolution of proteins [23]. A third method deals with optimizing sample solubility so as to ensure maximum protein coverage [24]. Indeed, the choice of detergent combination that is used during tissue homogenization has a large effect on protein coverage in 2DE experiments, and must be considered [25]. Taking all of these factors into consideration this proteomic technique is a powerful tool that can be utilized for various studies involving quantitative protein expression, isoform analysis, post-translational modification analysis, and biomarker discovery.

**Fig. (1):**
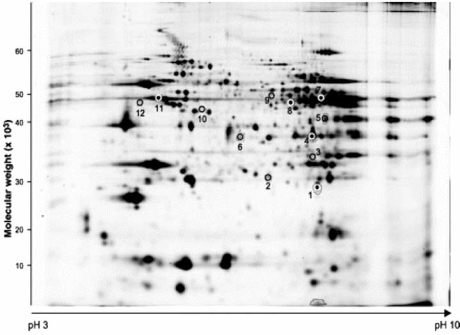
2D gel electrophoresis reveals proteins that were differentially expressed in PLM KO mice relative to wild-type controls. Spots of interest were excised and subjected to mass spectrometry analysis. Used with permission: Bell *et al*., Characterization of the phospholemman knockout mouse heart: depressed left ventricular function with increased Na-K-ATPase activity. Am J Physiol Heart Circ Physiol 2008; 294: H613-H621.

Recently, 2D-GE has been used to investigate several disease pathways in the heart [26]. One study examined a 20-fold increase in plasma nitric oxide (NO), a marker of NO synthesis, using 2D-GE and mass spectrometry in rat hearts subjected to sepsis and endotoxemia. The utility of such an approach is confirmed by the study which revealed an increase in the abundance of proteins involved in cellular metabolism, most notably ATP-synthase, Ubiquinol cytochrome-*c *reductase, and Elongation factor in a *Phospholemman *knockout (PLM KO) model of depressed contractile function (see Fig. **1**) [27]. The utility of 2D gels has also been recognized in the analysis of hearts that had been preconditioned to ischemia. Arrel *et al.* recently subjected rabbit ventricular myocytes to drugs which mimicked the effect of ischemic preconditioning and performed protein analysis that revealed significant alterations in proteins that played a role in mitochondrial energetics, chaperoning, and stress-responses [28]. These findings provided an unbiased view of potential pathways involved in preconditioning.

Gel based proteomic methods have also benefitted from the development of 2D DIGE (Differential Gel Electrophoresis) whereby up to three protein samples are fluorescently labeled with different fluorochromes and then subjected to gel electrophoresis, followed by scanning and analysis of the gel [29]. Such an approach has been put to use in the analysis of a model of dilated cardiomyopathy [30]. This gel-based method allowed for the identification of a consistent decrease in the phosphorylation of Tropomyosin (Tm). Given that this enzyme is a core component of the actin filament, regulating the interaction of actin and myosin, down-regulation of this protein could have implications for myocardial contractility. This study was highly successful in identifying a potential causes for this type cardiac disease, warranting further investigation in patient cohorts.

Although gel-based methods can be used to separate thousands of proteins and produce easily interpretable visual data, they possess some limitations. Membrane proteins are usually under-represented due to their poor solubility in the sample buffer and resolution in 2DE [31]. In general, gel-based techniques are biased towards the detection of highly abundant proteins and become less effective at detecting hydrophobic proteins and proteins with extreme pI and molecular weight [32]. Additionally, although significant progress has been in 2D gel methods, the lack of automation of extraction, digestion, and analysis of each spot still remains labour-intensive [31]. Alternatively, more groups are now shifting to a gel-free approach, which has been demonstrated to improve protein coverage.

### Gel-Free Separation

**Fig. (2):**
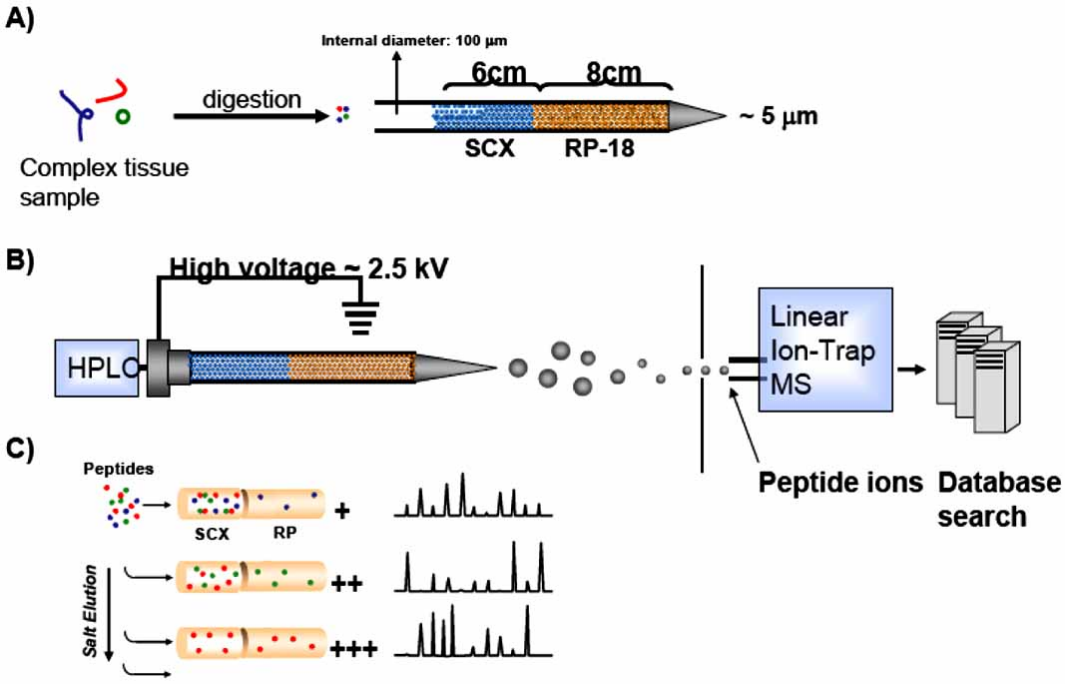
(**A**) Ventricular tissue taken from mice is subjected to trypsin digestion. Peptides are then separated on the basis of charge and hydrophobicity. (**B**) Eluted peptides are then electrosprayed into a Linear Ion-Trap Mass Spectrometer *via* electrospray ionization (ESI). The resultant mass spectra are matched to proteins using database algorithms. (**C**) Increasing salt concentrations, or “salt bumps”, elute peptides from the LC columns providing enhanced peptide separation. *Used with Permission:* Chugh et al., Large-scale studies to identify biomarkers for heart disease: a role for proteomics? Expert Opin Med Diagn 2009; 3(2): 133-141.

Gel-free systems are being increasingly utilized for proteomic-based experiments. A common gel-free technique is the Multidimensional Protein Identification Technology (MudPIT), pioneered by the Yates group, which utilizes multi-dimensional liquid chromatography as a separation method analogous to 2DE coupled with tandem mass spectrometry (MS/MS) [31, 33]. The technique uses biphasic capillary columns packed with strong cation exchange material (SCX) and reverse phase material (RP) allowing for the separation of protein samples by charge and hydrophobicity [33], (see Fig. **2**).

The main advantages of this method over 2D gel approaches are that this approach involves reduced sample handling and thus less potential for sample loss since the column is placed in-line with a tandem mass spectrometer to allow analysis of peptides as they are eluted off the column. For example, this strategy has been applied to the characterization of the yeast proteome [33], several major mouse organs [34-36], embryonic stem cells [37], cytosol [36, 38], mitochondria [39], nucleus [40], and nucleolus [41].

Similarly, Gramolini *et al.* performed gel-free shotgun sequencing of proteolytic digests of ventricular tissue extracts from phospholamban (PLN-R9C) mutant mice exhibiting dilated cardiomyopathy using high performance multidimensional liquid capillary-scale chromatography (HPLC) coupled to automated data-dependent tandem mass spectrometry (LTQ linear ion trap mass spectrometer) [5]. Of the 6190 proteins identified, 593 were revealed to be differentially expressed between wild-type and R9C hearts. Gel-free separation can now be coupled with sophisticated proteomic technology to allow for direct detection of proteins from complex mixtures. Jullig *et al.* coupled LC-MS with iTRAQ (Isobaric Tag for Relative and Absolute Quanitification) technology in their identification of proteins involved in cardiac mitochondria from diabetic hearts [42]. In these experiments, proteins from samples are covalently tagged at the N-terminus and at amine sidechains with different tags allowing for detection, were pooled together and analyzed by LC-MS to determine which protein sequences are present. This analysis was useful in uncovering 65 differentially regulated proteins in the disease state, emphasizing the involvement of the mitochondria in the progression of diabetes.

### Ionization of the Proteome

In addition to the advances that have been made in gel-based and gel-free systems, sophistication of additional platforms has revolutionized the field of clinical proteomics. MALDI-TOF (Matrix-Assisted Laser Desorption/Ionization Time-Of-Flight) mass spectrometry has undergone rapid changes and improvements in recent years, making it a desirable tool for studying the cardiac proteome. This approach utilizes nanosecond laser pulses which vaporize specimens that have been dried and spotted on a target place with a light-absorbing matrix molecule [43]. This platform is advantageous over other MS ionization methods because of its high sensitivity to peptides and proteins of low molecular weight. In addition to the increased sensitivity and mass accuracy, MALDI MS allows for rapid analysis. Tissue sections and whole cells can be directly analyzed, whereas traditional analysis methods require the homogenization and purification of tissue [44]. SELDI (Surface-Enhanced Laser Desorption/Ionization) analysis is a refinement of MALDI in which the metal surface is coated with a specific chemical that takes advantage of antibodies, ionic interactions and hydrophobicity to preselect a subset proteins to be preselected before MS analysis [45]. This is especially useful when the proteome area of interest is already known. For example, immunoaffinity enrichment with different specific Tn monoclonal antibodies were used to validate elevated cTnI as a signature for myocardial infarction, by way of SELDI-TOF MS [45]. This approach has also been used successfully to identify predictive plasma biomarkers of left ventricular remodeling after acute myocardial infarction. This analysis revealed elevated levels of post-translational variants of Hpα1 (α1-chain of haptoglobin) in plasma of patients with left ventricular remodeling [46].

MALDI-MS has evolved not only as a powerful discovery tool but more recently as an imaging system. MALDI-IMS (MALDI Imaging Mass Spectrometry) involves the microspotting of tissue samples within a MALDI matrix followed by the acquisition of spectra by mass spectrometry at discrete locations on a predefined “grid” [47]. Each of these spots can be represented as a “pixel” representative of a two dimensional of a tissue sample. They can then be combined to provide a spatial representation of the distribution of a particular protein or molecule [47]. Although this system has yet to be employed for the heart to our knowledge, it has the potential to become an extremely powerful tool for understanding protein expression in pathological states. Evidently, both gel-based and gel-free systems have contributed significantly to the field of clinical proteomics. A brief summary of recent proteomics studies is provided to emphasize the contributions of mass spectrometry based platforms to the investigation of cardiovascular disease (see Table **1**).

## 4. TOOLS FOR VALIDATION

Most biomarker discovery tools such as MS-based analysis still require validation of candidate markers in clinical samples. This calls for a highly specific, high throughput systems that can verify specific protein levels in a large number of samples. Conventional experimental tools, such as protein microarrays and ELISA, rely on antibodies and immunoassays designed to detect specific proteins or peptides. Forward phase protein arrays (FPAs) are widely used in research to quantify the relative abundance of antigens in samples. Antibodies are immobilized on the surface of a chip, which then specifically bind protein antigens from serum samples, cellular lysate, or urine samples. The bound analytes are then detected by the application of a second labeled antibody, usually by means of immunofluorescence [48]. Alternatively, reverse protein arrays (RPAs) do not require immobilized antibodies. Instead, tissue samples are spotted onto a chip’s surface and probed with various antibodies to detect relative abundance [49]. Although RPAs are not commonly used in the assessment of cardiac disease, they have been successfully applied to analyze biomarkers of cancer [50]. Protein microarrays are advantageous in that they allow high-throughput screening in a time-effective manner. ELISA tests are another commonly employed technique used to validate biomarkers. Here, analytes from samples are captured by immobilized antibodies fixed to a plate surface. An enzyme-linked detecting antibody is added, which is then activated by the addition of a substrate that changes colour or fluoresces when catalyzed. Since detection of the analyte is based on the proportion of catalyzed substrate, this indirect method amplifies signal and is highly sensitive. Consequently, proteins in the picomolar to nanomolar range can be effectively quantified, making it suitable for clinical applications [48]. However, FPAs, RPAs, and ELISAs all rely on obtaining highly-specific antibodies against known target proteins. Currently, there are limited numbers of highly reliable antibodies available to a small proportion of the proteome. Also, antibody-based systems inevitably suffer from cross-reactivity of antibodies and interference of protein-antibody interactions [51].

Multiple reaction monitoring mass spectrometry (MRM-MS) may have greater potential as a general scientific approach. Traditional MS runs acquire a full-scan of peptides within a defined mass range, and generally detect higher abundance peptides, resulting in a ‘parent ion’. These peptides can trigger a second MS scan where the peptides are fragmented and acquired (daughter ion). The combination of the specific parent mass and the unique fragment ion is used to selectively identify a protein or peptide. As an alternative to this ‘data-dependent’ platform, MRM-MS can be programmed to target a specific peptide subpopulation by monitoring for only selected precursors representative of a protein of interest. This provides high selectivity by monitoring several transitions for a particular peptide by chromatographic elution [52]. However, the most important advantage of this approach is that this method does not require an antibody, only knowledge of the parent/daughter ion signatures. As such, numerous quantitative assays can be developed and monitored for proteins where no reagents currently exist. MRM has been applied successfully to quantify protein expression [53], to elucidate cellular signaling pathways in the EGFR network [52], and to discover serum biomarkers to assess severity of rheumatoid arthritis [54]. 

In addition to the advantages of proteomic-based studies, there are challenges which may stand in the way of achieving optimal results. Sample complexity is a major concern that must be taken into account in all proteomic based studied. Cardiac tissue samples, for example, may contain a dynamic range in protein abundance, with sarcomeric proteins being present in much higher quantities than other cardiac resident tissue proteins. This complexity is problematic for mass spectrometry analysis. Since peptides are selected for analysis at the collision cell of the mass spectrometer, peptides present in high amounts interfere with accurate identification of lower abundance peptides. Thus, there may be a misrepresentation of peptide sequences within a given sample. In their study, Kuster *et al.* reported that potentially hundreds of thousands of peptides may be present following digestion of a sample, which could lead to undersampling of peptide ions [55]. This overwhelming amount of peptide sequences arises predominantly from the large number of unique protein sequences owing to post-transcriptional modifications, processing of proteolytic segments, and alternative splicing of peptide sequences, as well as genetic variation among individuals. To address this issue, techniques must be set in place to reduce sample complexity [56]. In cardiac samples, for example, pre-fractionation is one such procedure that shows promise in minimizing sample complexity as it analyzes various cellular fractions independently, after which abundance levels can be compiled in each separate fraction [5, 57, 58]. The utility of this approach was confirmed by Havugimana *et al.* who performed a proteomic analysis of cardiac samples that were pre-fractionated and those that were not. They discovered a marked increase in protein coverage in samples that had been pre-fractionated versus those that had not [59]. Sample complexity is also an issue of concern in blood. Albumin and immunoglobins, namely IgG, represent 99% large portion of the proteome [60]. Because there are myriads of undiscovered proteins in blood that could serve as biomarkers of disease, albumin and IgG must be depleted prior to in depth analysis. To date, various techniques have been described to achieve this goal [61, 62]. Another potential challenge of proteomic based experiments lay in sample selection. Many proteomic studies which analyze levels of proteins in blood make the assumption that there is a similar expression pattern in the diseased tissue. This hypothesis does not always hold true, since a protein biomarker has been significantly diluted from its journey from diseased tissue to body fluids such as blood [63]. However, as technological improvements in mass spectrometry and pre-fractionation continue to improve sensitivity and specificity and overcome these challenges novel biomarkers of disease will be identified with greater confidence. 

## 5. MYOCARDIAL ISCHEMIA

Proteomic platforms have also been exploited in order to gain insight on to disease pathways involved in the progression of myocardial ischemia. During myocardial ischemia the normal balance between oxygen supply and demand is compromised, with the latter dominating. Taking into consideration the fact that the heart requires a continuous supply of oxygen to carry out normal function, a decrease in the oxygen available could lead to myocardial tissue damage.

Patients who have myocardial ischemia often present with angina, dyspnea, or diaphoresis which would lead a clinician to pursue further diagnostic tests. Investigations may be performed at rest or upon exertion, to ascertain the severity of the disease. Traditionally, exercise testing in conjunction with electrocardiography has been a standard test for the initial assessment of patients suspected of having some degree of ischemia. However, new clinical diagnostics are needed to complement these tests to improve the prognosis of this disease state. 

Proteins used as markers can be part of a signaling pathway activated during disease progression, or simply an unrelated protein that is overexpressed in the disease state. Nevertheless, both groups of proteins serve an important purpose in the diagnosis of ischemia. Eugene Braunwald pointed out that biomarkers can be classified into six groups: inflammation, oxidative stress, extracellular-matrix remodeling, neurohormones, myocyte injury, and myocyte stress. Markers of myocardial ischemia generally fall into the group of myocyte injury, such as ischemia-modified albumin [64, 65], unbound free fatty acid [66], NT-proBNP/BNP [67-69], and IL-6 [70]. Exploration into various other categories, such as inflammatory markers or proteins involved in remodeling, could offer a new and innovative means to diagnose this disease process in the early stages. Proteomic strategies are now being used to uncover proteins that would have not been identifiable in the past.

During myocardial ischemia, myocyte stress leads to mitochondrial damage. This hallmark feature is beginning to be exploited to determine additional markers of this disease state. Weiss *et al.* describe the phenomenon of Mitochondrial Permeability Transition (MPT), occurring during ischemia/reperfusion, during which there is irreversible injury to the mitochondria [71]. Here, an increase in the permeability of specialized mitochondrial pores leads to swelling of the mitochondrial matrix and subsequent rupture of the outer membrane with a corresponding release of apoptotic proteins. However, there is much to be discovered in relation to activated protein pathways and other key post-translational modifications that occur during this disease state. In an attempt to fill this gap, Chen *et al.* used a proteomic approach to determine which proteins were differentially regulated in ischemic hearts [72]. Specifically, proteins were first tagged with fluorescent dyes and then resolved on a 2D gel, allowing for visual detection of alterations in protein expression. Spots of interest were then excised from the gel and underwent subsequent analysis using liquid chromatography mass spectrometry. This approach allowed for the discovery of the mitochondrial protein, aldehyde dehydrogenase type 2 (ALDH2), a cardioprotective protein. In a similar endeavor, Jacquet *et al.* used a similar approach to improve clinical diagnosis of Acute Myocardial Infarctions (AMI) [73]. Here, gel electrophoresis was used to determine which proteins were abundant in cardiac samples taken from mice exposed to ischemic conditions. In addition to previously established biomarkers, this group showed cardiac myosin binding protein C as a potential biomarker of AMI. Of interest, the expression of this protein far exceeded that of certain previously established biomarkers of AMI, emphasizing the utility of this protein for clinical diagnosis. Indeed, this study has set the stage for further analysis of this protein in patient cohort samples as well as its involvement in pathways following myocardial infarction.

Blood contains a repository of unexplored biomarkers which, if identified, have the potential to revolutionize the treatment of various disease forms [74]. When subjected to stress, as in myocardial ischemia, cells often secrete specific proteins into the blood which reflect the physiological state. Highly impactful are proteomic experiments performed directly on blood samples taken from human patients who suffer from ischemia. Those proteins that would be found to be differentially expressed in diseased blood would be further pursued as potential biomarkers. This approach has been applied effectively to samples taken from patients with inducible ischemia [75]. Following resolution by liquid chromatography, mass spectrometry was performed to determine which proteins had altered expression in this disease state. Six metabolites were found to possess significant alteration during myocardial ischemia. Of these, 193 and MET 121 to be increased during myocardial ischemia, whereas MET 200, gamma aminobutyric acid, uric acid, and citric acid were decreased in the diseased state. As discussed previously, examination of proteins in blood presents many technical challenges that need to be overcome to arrive at accurate results. Furthermore, the assumption that protein expression patterns in tissue and blood are always concordant, has been a point of contention [76]. Nevertheless, Zhang *et al.* demonstrated that, in fact, expression patterns in both samples are similar. [77]. Here, they show that there may not be a linear relationship between changes in protein expression in tissue and alterations in blood [76]. By examining glycosylation patterns of peptides, using mass spectrometric analysis, they were able to confirm that many proteins detected in tissue were also present in blood. However, biomarkers showing promise of being used in a clinical setting should be analyzed in tissue samples to ensure concordant expression patterns, thereby providing a powerful marker of the disease state.

Well established as a major cause of myocardial ischemia is an increase in oxygen demand in the presence of fixed coronary vessel narrowing. Physical obstruction can be attributed to the formation of atherosclerotic plaques within these arteries. As low-density lipoproteins (LDL) and other inflammatory molecules accumulate in the coronary arteries, plaques will often destabilize which can lead to a rupture and thrombosis. Given that atherosclerosis is the leading cause of myocardial ischemia, various proteomic-based studies have analyzed atherosclerotic plaques. Specifically, proteins that have significant alterations in expression in such tissue samples could be further studied as potential markers in patients who have ischemia. For instance, a proteomic strategy was utilized in the determination of proteins that were differentially expressed in atherosclerotic tissue samples, relative to controls [78]. Notably, actin, tropomyosin-like proteins, and two glycoproteins were identified in this study. A subsequent analysis of patients who had coronary artery disease was performed whereby coronary arteries from diseased patients and matched controls were used [79]. Their proteomic platform, involving 2D-gel electrophoresis followed by mass spectrometric analysis, revealed ferritin light chain to be overexpressed. This result was highly significant as this protein likely contributes to the progression of myocardial ischemia. Within atherosclerotic plaques, the generation of reactive oxygen species has been studied in depth as such radicals modify the lipids and proteins of Low Density Lipoproteins. Ferritin light chain may act by altering the generation of reactive oxygen species, thereby regulating the growth of atherosclerotic plaques. This promising biomarker would benefit from validation in additional clinical studies which include patients with signs and symptoms of early stage myocardial ischemia. 

## 6. CONCLUSION

Given the overall health burden felt by cardiomyopathy on a global scale, there still remains an urgent need for new and innovative therapies for these diseases. Proteomic-based studies may be able to provide these markers of disease and new therapeutic avenues that may one day be translated into the clinical setting where they will be used regularly in the diagnosis and treatment of cardiac disease. Obviously, the transition from bench to bedside is not an easy one, and must be facilitated by subsequent, carefully designed, clinical trials.

## Figures and Tables

**Table 1.** Recent Biomarker and Disease Pathway Elucidation with the Advancement in Various Proteomic Based Technologies

Biology	Discovery	Proteomic Technique	Species	Reference
Nitrite mediated cardioprotection	Involvement of following mitochondrial proteins: PDIA3, COQ9, ALDH2,	MALDI-TOF MS	Rat	[80]
Hypertension induced cardiomyopathy	Decrease in expression of metabolic proteins: adenylate kinase 1, creatine kinase-M, lactate dehydrogenase	2DE, Orbitrap MS	Mouse	[81]
Protein expression in the aging ventricle	Elucidation of protein networks affected: carbohydrate metabolism, cell morphology, cell assembly Significant fluctuations in metabolic proteins	iTRAQ	Rat	[82]
Adrenaline and/or reactive oxygen species mediated cardiomyopathy	Increase in expression of myosin regulatory light chain 2 in both groups, decrease in proteins involved in energetic metabolism in both groups (electron transfer flavoprotein beta subunit)	MALDI-TOF	Rat	[83]
Differentiating protein expression in failing and non-failing hearts	Identification of known and novel proteins involved in the progression to overt failure, with a corresponding correlation to expression patterns seen in the wild-type cardiac transcriptome, proteome, and phosphoproteome	Reverse Phase Gel-Free Liquid Chromatography	Human	[84]
Proteomic analysis of chronic heart failure	Discovery of heat shock proteins, endoplasmic reticulum stress proteins, oxidative stress proteins, and metabolic enzymes involved in progression to CHF	2DE	Rat	[10]
Proteomic alterations in cardiac disease in frataxin knockout mice	Decreases in expression of iron dependent complexes in the mitochondrial apparatus, with an increase in citric acid cycle enzymes and catabolic enzymes	2DE	Mouse	[85]
Proteomic analysis of hyperdynamic mouse hearts	Identification of differential regulation of myosin light chain isoforms, and post-translational modifications.	2DE, MALDI TOF, LC-MS/MS	Mouse	[86]
Differential expression of ferritin light chain in coronary atheroscelerosis	Increased expression of ferritin light chain in diseased coronary arteries, confirming that there is an increase in iron during CAD	2DE	Human	[79]
Isolation of cardiac troponins from biological samples	A unique method for the extraction of cardiac troponins from the left ventricle of patients with cardiovascular disease is described	Affinity chromatography, MALDI-TOF	Human	[87]
Proteomic profiling of chronic heart failure	Discovery of upregulation in HSP70 in patients with arrhythmogenic right ventricular cardiomyopathy relative to control samples	2DE, MS	Human/Rat	[9]
Investigation of post-translational modifications of cardiac troponin I	Proteomic approach used to understand the complex interplay between phosphorylation and proteolysis of cardiac troponin I, to strengthen its role as a biomarker	ESI/FTMS	Human	[88]
Proteomic analysis of diabetic myocardial proteome	Profiling of the diseased cardiac proteome allowed for the analysis of free-radical production as well as antioxidant defense mechanisms.	2DE, MALDI-TOF MS	Rat	[89]
Proteomic analysis of ischemia/reperfusion model	Validated isolated organ perfusion as a potential diagnostic tool for biomarker discovery	2DE LC-MS/MS	Rat	[4]
Proteomic analysis of ischemia/reperfusion model	Identified 25 mitochondrial proteins involved in mitochondrial respiratory chain and energy metabolism differentially expressed in ischemia-reperfusion hearts	2DE MALDI-TOF	Rabbit	[3]
Proteomic analysis of myocardial infarction	Gel-free analysis of cardiac troponin isoforms from blood samples of patients with acute myocardial infarctions	SELDI-TOF	Human	[2]
Clinical detection of BNP in patients with congestive heart failure	Developed a surface affinity chip to enhance enrichment, separation, and detection of BNP in human plasma.	MALDI-TOF	Human	[8]
Innovative proteomic technique for the analysis of myocardial hibernation	Demonstrated improvement of a label-free LC-Orbitrap method over a parallel study using 2DE for expression profiling of myocardial mitochondrial proteins	LC-Orbitrap	Pig	[90]
Proteomic analysis of rat model of heart failure	Identified and quantified changes in cardiac mitochondrial proteins	iTRAQ MuDPIT MS/MS	Rat	[42]
Comparative proteomic profiling of a rat model of heart failure resulting from cardiac hypertrophy	Changes in 33 left ventricular mitochondrial proteins in pre-hypertensive/hypertensive stages of cardiac hypertrophy	2DE MALDI-TOF/TOF (tandem MS)	Rat	[91]
Identification of Hpα1 variants involved in the progression to heart failure	Post-translational variants of Hpα1 as plasma biomarkers for left ventricular remodeling from patients with acute myocardial infarctions	SELDI-TOF	Human	[46]
Comparative proteomic profiling of a mouse model of dilated cardiomyopathy	Large-scale shotgun analysis revealed 593 differentially regulated proteins in mice with dilated cardiomyopathy, including key apoptotic proteins	LC-MS, MuDPIT	Mouse	[5]

## ABBREVIATIONS

CTN I: = Cardiac Troponin I
CK-MB: = Creatine Kinase-Myoglobin
2D-GE: = Two Dimensional-Gel Electrophoresis
*p*I: = Isoelectric Point
DIGE: = Differential Gel Electrophoresis
Tm: = Tropomyosin
PEDF: = Pigment-Epithelium Derived Factor
MudPIT: = Multidimensional Protein Identification Technology
MS: = Mass Spectrometry
SCX: = Strong Cation Exchange
RP: = Reverse Phase
PLN-R9C: = Phospholamban- Arginine to Cysteine Mutation
HPLC: = High Performance Liquid Chromatography
iTRAQ: = Isobaric Tag for Relative and Absolute Quanitification
LC-MS: = Liquid Chromatography-Mass Spectrometry
MALDI-TOF: = Matrix-Assisted Laser Desorption-Ionization Time-Of-Flight
SELDI: = Surface-Enhanced Laser Desorption/Ionization
TOF: = Time of Flight Surface-Enhanced Laser Desorption/Ionization
Hpα1: = α1-chain of haptoglobin
IMS: = Imaging Mass Spectrometry
PDIA3: = Protein Disulfide Isomerase family A, member 3
COQ9: = Coenzyme Q9
ALDH2: = Aldehyde Dehydrogenase 2
HSP70: = Heat Shock Protein 70
ELISA: = Enzyme Linked Immunosorbent Assay
FPA: = Forward Protein Assay
RPA: = Reverse Protein Assay
MRM-MS: = Multiple reaction monitoring mass spectrometry
EGFR: = Epidermal Growth Factor Receptor
BNP: = Brain Naturietic Peptide
IL-6: = Interleukin-6
MPT: = Mitochondrial Permeability Transition
AMI: = Acute Myocardial Infarction
LDL: = Low Density Lipoprotein
NO: = Nitric Oxide
PLM-KO: = Phospholemman-Knockout

## References

[1] Kislinger T, Emili A (2003). Going global: protein expression profiling using shotgun mass spectrometry. Curr Opin Mol Ther.

[2] Peronnet E, Becquart L, Poirier F, Cubizolles M, Choquet-Kastylevsky G, Jolivet-Reynaud C (2006). SELDI-TOF MS analysis of the Cardiac Troponin I forms present in plasma from patients with myocardial infarction. Proteomics.

[3] Kim N, Lee Y, Kim H (2006). Potential biomarkers for ischemic heart damage identified in mitochondrial proteins by comparative proteomics. Proteomics.

[4] Koomen JM, Wilson CR, Guthrie P, Androlewicz MJ, Kobayashi R, Taegtmeyer H (2006). Proteome analysis of isolated perfused organ effluent as a novel model for protein biomarker discovery. J Proteome Res.

[5] Gramolini AO, Kislinger T, Alikhani-Koopaei R (2008). Comparative proteomics profiling of a phospholamban mutant mouse model of dilated cardiomyopathy reveals progressive intracellular stress responses. Mol Cell Proteomics.

[6] Weekes J, Wheeler CH, Yan JX (1999). Bovine dilated cardiomyopathy: proteomic analysis of an animal model of human dilated cardiomyopathy. Electrophoresis.

[7] Buscemi N, Murray C, Doherty-Kirby A, Lajoie G, Sussman MA, Van Eyk JE (2005). Myocardial subproteomic analysis of a constitutively active Rac1-expressing transgenic mouse with lethal myocardial hypertrophy. Am J Physiol.

[8] Chen YQ, Bi F, Wang SQ, Xiao SJ, Liu JN (2008). Porous silicon affinity chips for biomarker detection by MALDI-TOF-MS. J Chromatogr.

[9] Wei YJ, Huang YX, Shen Y (2009). Proteomic analysis reveals significant elevation of heat shock protein 70 in patients with chronic heart failure due to arrhythmogenic right ventricular cardiomyopathy. Mol Cell Biochem.

[10] Cieniewski-Bernard C, Mulder P, Henry JP (2008). Proteomic analysis of left ventricular remodeling in an experimental model of heart failure. J Proteome Res.

[11] Morrow DA, de Lemos JA (2007). Benchmarks for the assessment of novel cardiovascular biomarkers. Circulation.

[12] Bignami E, Landoni G, Crescenzi G (2009). Role of cardiac biomarkers (troponin I and CK-MB) as predictors of quality of life and long-term outcome after cardiac surgery. Ann Card Anaesth.

[13] McGregor E, Dunn MJ (2003). Proteomics of heart disease. Hum Mol Gen.

[14] O'Farrell PH (1975). High resolution two-dimensional electrophoresis of proteins. J Biol Chem.

[15] McGregor E, Dunn MJ (2006). Proteomics of the heart: unraveling disease. Circ Res.

[16] Lang SC, Elsasser A, Scheler C (2006). Myocardial preconditioning and remote renal preconditioning--identifying a protective factor using proteomic methods?. Basic Res Cardiol.

[17] Yan L, Vatner DE, Kim SJ, Ge H (2005). Autophagy in chronically ischemic myocardium. Proc Natl Acad Sci USA.

[18] Faber MJ, Dalinghaus M, Lankhuizen IM (2005). Proteomic changes in the pressure overloaded right ventricle after 6 weeks in young rats: correlations with the degree of hypertrophy. Proteomics.

[19] Shen X, Zheng S, Thongboonkerd V (2004). Cardiac mitochondrial damage and biogenesis in a chronic model of type 1 diabetes. Am J Physiol Endocrinol Metab.

[20] Gorg A, Weiss W, Dunn MJ (2004). Current two-dimensional electrophoresis technology for proteomics. Proteomics.

[21] Barnouin K (2004). Two-dimensional gel electrophoresis for analysis of protein complexes. Methods Mol Biol.

[22] Rabilloud T (2002). Two-dimensional gel electrophoresis in proteomics: old, old fashioned, but it still climbs up the mountains. Proteomics.

[23] Righetti PG, Castagna A, Antonioli P, Boschetti E (2005). Prefractionation techniques in proteome analysis: the mining tools of the third millennium. Electrophoresis.

[24] Guo Y, Fu Z, Van Eyk JE (2007). A proteomic primer for the clinician. Proc Am Thorac Soc.

[25] Stanley BA, Neverova I, Brown HA, Van Eyk JE (2003). Optimizing protein solubility for two-dimensional gel electrophoresis analysis of human myocardium. Proteomics.

[26] Robichaud S, Lalu M, Udenberg T, Schulz R, Sawicki G (2009). Proteomics analysis of changes in myocardial proteins during endotoxemia. J Proteomics.

[27] Bell JR, Kennington E, Fuller W (2008). Characterization of the phospholemman knockout mouse heart: depressed left ventricular function with increased Na-K-ATPase activity. Am J Physiol.

[28] Arrell DK, Elliott ST, Kane LA (2006). Proteomic analysis of pharmacological preconditioning: novel protein targets converge to mitochondrial metabolism pathways. Circ Res.

[29] Marouga R, David S, Hawkins E (2005). The development of the DIGE system: 2D fluorescence difference gel analysis technology. Anal Bioanal Chem.

[30] Warren CM, Arteaga GM, Rajan S, Ahmed RP, Wieczorek DF, Solaro RJ (2008). Use of 2-D DIGE analysis reveals altered phos-phorylation in a tropomyosin mutant (Glu54Lys) linked to dilated cardiomyopathy. Proteomics.

[31] Kline KG, Wu CC (2009). MudPIT analysis: application to human heart tissue. Methods Mol Biol.

[32] Edwards AV, White MY, Cordwell SJ (2008). The role of proteomics in clinical cardiovascular biomarker discovery. Mol Cell Proteomics.

[33] Washburn MP, Wolters D, Yates JR 3rd (2001). Large-scale analysis of the yeast proteome by multidimensional protein identification technology. Nat Biotechnol.

[34] Cutillas PR, Vanhaesebroeck B (2007). Quantitative profile of five murine core proteomes using label-free functional proteomics. Mol Cell Proteomics.

[35] Kislinger T, Cox B, Kannan A (2006). Global survey of organ and organelle protein expression in mouse: combined proteomic and transcriptomic profiling. Cell.

[36] Shi R, Kumar C, Zougman A (2007). Analysis of the mouse liver proteome using advanced mass spectrometry. J Proteome Res.

[37] Graumann J, Hubner NC, Kim JB (2008). Stable isotope labeling by amino acids in cell culture (SILAC) and proteome quantitation of mouse embryonic stem cells to a depth of 5,111 proteins. Mol Cell Proteomics.

[38] Shin YK, Lee HJ, Lee JS, Paik YK (2006). Proteomic analysis of mammalian basic proteins by liquid-based two-dimensional column chromatography. Proteomics.

[39] Mootha VK, Bunkenborg J, Olsen JV (2003). Integrated analysis of protein composition, tissue diversity, and gene regulation in mouse mitochondria. Cell.

[40] Schirmer EC, Florens L, Guan T, Yates JR, 3rd Gerace L (2003). Nuclear membrane proteins with potential disease links found by subtractive proteomics. Science.

[41] Andersen JS, Lam YW, Leung AK (2005). Nucleolar proteome dynamics. Nature.

[42] Jullig M, Hickey AJ, Chai CC (2008). Is the failing heart out of fuel or a worn engine running rich? A study of mitochondria in old spontaneously hypertensive rats. Proteomics.

[43] Hortin GL (2006). The MALDI-TOF mass spectrometric view of the plasma proteome and peptidome. Clin Chem.

[44] Reyzer ML, Caprioli RM (2005). MALDI mass spectrometry for direct tissue analysis: a new tool for biomarker discovery. J Proteome Res.

[45] Petricoin EF, Ardekani AM, Hitt BA (2002). Use of proteomic patterns in serum to identify ovarian cancer. Lancet.

[46] Pinet F, Beseme O, Cieniewski-Bernard C (2008). Predicting left ventricular remodeling after a first myocardial infarction by plasma proteome analysis. Proteomics.

[47] Cornett DS, Reyzer ML, Chaurand P, Caprioli RM (2007). MALDI imaging mass spectrometry: molecular snapshots of biochemical systems. Nat Methods.

[48] Arab S, Gramolini AO, Ping P (2006). Cardiovascular proteomics: tools to develop novel biomarkers and potential applications. J Am Coll Cardiol.

[49] Zong Y, Zhang S, Chen HT, Zong Y, Shi Y (2007). Forward-phase and reverse-phase protein microarray. Method Mol Biol.

[50] Paweletz CP, Charboneau L, Bichsel VE (2001). Reverse phase protein microarrays which capture disease progression show activation of pro-survival pathways at the cancer invasion front. Oncogene.

[51] Talapatra A, Rouse R, Hardiman G (2002). Protein microarrays: challenges and promises. Pharmacogenomics.

[52] Wolf-Yadlin A, Hautaniemi S, Lauffenburger DA, White FM (2007). Multiple reaction monitoring for robust quantitative proteomic analysis of cellular signaling networks. Proc Natl Acad Sci USA.

[53] Kirkpatrick DS, Gerber SA, Gygi SP (2005). The absolute quantification strategy: a general procedure for the quantification of proteins and post-translational modifications. Methods.

[54] Liao H, Wu J, Kuhn E (2004). Use of mass spectrometry to identify protein biomarkers of disease severity in the synovial fluid and serum of patients with rheumatoid arthritis. Arthritis Rheum.

[55] Kuster B, Schirle M, Mallick P, Aebersold R (2005). Scoring proteomes with proteotypic peptide probes. Nat Rev.

[56] Gerszten RE, Accurso F, Bernard GR (2008). Challenges in translating plasma proteomics from bench to bedside: update from the NHLBI Clinical Proteomics Programs. Am J Physiol.

[57] Kislinger T, Rahman K, Radulovic D, Cox B, Rossant J, Emili A (2003). PRISM, a generic large scale proteomic investigation strategy for mammals. Mol Cell Proteomics.

[58] Sandhu C, Connor M, Kislinger T, Slingerland J, Emili A (2005). Global protein shotgun expression profiling of proliferating mcf-7 breast cancer cells. J Proteome Res.

[59] Havugimana PC, Wong P, Emili A (2007). Improved proteomic discovery by sample pre-fractionation using dual-column ion-exchange high performance liquid chromatography. J Chromatogr.

[60] Horvatovich P, Govorukhina N, Bischoff R (2006). Biomarker discovery by proteomics: challenges not only for the analytical chemist. Analyst.

[61] Seam N, Gonzales DA, Kern SJ, Hortin GL, Hoehn GT, Suffredini AF (2007). Quality control of serum albumin depletion for proteomic analysis. Clin Chem.

[62] Baussant T, Bougueleret L, Johnson A (2005). Effective depletion of albumin using a new peptide-based affinity medium. Proteomics.

[63] Schiess R, Wollscheid B, Aebersold R (2009). Targeted proteomic strategy for clinical biomarker discovery. Mol Oncol.

[64] Sinha MK, Gaze DC, Tippins JR, Collinson PO, Kaski JC (2003). Ischemia modified albumin is a sensitive marker of myocardial ischemia after percutaneous coronary intervention. Circulation.

[65] Quiles J, Roy D, Gaze D (2003). Relation of ischemia-modified albumin (IMA) levels following elective angioplasty for stable angina pectoris to duration of balloon-induced myocardial ischemia. Am J Cardiol.

[66] Kleinfeld AM, Prothro D, Brown DL, Davis RC, Richieri GV, DeMaria A (1996). Increases in serum unbound free fatty acid levels following coronary angioplasty. Am J Cardiol.

[67] de Lemos JA, Morrow DA, Bentley JH (2001). The prognostic value of B-type natriuretic peptide in patients with acute coronary syndromes. N Engl J Med.

[68] James SK, Lindahl B, Siegbahn A (2003). N-terminal pro-brain natriuretic peptide and other risk markers for the separate prediction of mortality and subsequent myocardial infarction in patients with unstable coronary artery disease: a Global Utilization of Strategies To Open occluded arteries (GUSTO)-IV substudy. Circulation.

[69] Dagianti A, Penco M, Agati L (1995). Stress echocardiography: comparison of exercise, dipyridamole and dobutamine in detecting and predicting the extent of coronary artery disease. J Am Coll Cardiol.

[70] Ikonomidis I, Athanassopoulos G, Lekakis J (2005). Myocardial ischemia induces interleukin-6 and tissue factor production in patients with coronary artery disease: a dobutamine stress echocardiography study. Circulation.

[71] Weiss JN, Korge P, Honda HM, Ping P (2003). Role of the mitochondrial permeability transition in myocardial disease. Circ Res.

[72] Chen CH, Budas GR, Churchill EN, Disatnik MH, Hurley TD, Mochly-Rosen D (2008). Activation of aldehyde dehydrogenase-2 reduces ischemic damage to the heart. Science.

[73] Jacquet S, Yin X, Sicard P (2009). Identification of cardiac myosin binding protein C as a candidate biomarker of myocardial infarction by proteomic analysis. Mol Cell Proteomics.

[74] Anderson NL, Anderson NG (2002). The human plasma proteome: history, character, and diagnostic prospects. Mol Cell Proteomics.

[75] Sabatine MS, Liu E, Morrow DA (2005). Metabolomic identification of novel biomarkers of myocardial ischemia. Circulation.

[76] Beretta L (2007). Proteomics from the clinical perspective: many hopes and much debate. Nat Method.

[77] Zhang H, Liu AY, Loriaux P (2007). Mass spectrometric detection of tissue proteins in plasma. Mol Cell Proteomics.

[78] Stastny J, Fosslien E, Robertson AL Jr (1986). Human aortic intima protein composition during initial stages of atherogenesis. Atherosclerosis.

[79] You SA, Archacki SR, Angheloiu G (2003). Proteomic approach to coronary atherosclerosis shows ferritin light chain as a significant marker: evidence consistent with iron hypothesis in atherosclerosis. Physiol Genomics.

[80] Perlman DH, Bauer SM, Ashrafian H (2009). Mechanistic insights into nitrite-induced cardioprotection using an integrated metabolomic/proteomic approach. Circ Res.

[81] Zlatkovic J, Arrell DK, Kane GC, Miki T, Seino S, Terzic A (2009). Proteomic profiling of KATP channel-deficient hypertensive heart maps risk for maladaptive cardiomyopathic outcome. Proteomics.

[82] Grant JE, Bradshaw AD, Schwacke JH, Baicu CF, Zile MR, Schey KL (2009). Quantification of Protein Expression Changes in the Aging Left Ventricle of Rattus norvegicus. J Proteome Res.

[83] Costa VM, Silva R, Tavares LC (2009). Adrenaline and reactive oxygen species elicit proteome and energetic metabolism modifications in freshly isolated rat cardiomyocytes. Toxicology.

[84] Ruse CI, Tan FL, Kinter M, Bond M (2004). Intregrated analysis of the human cardiac transcriptome, proteome and phosphoproteome. Proteomics.

[85] Sutak R, Xu X, Whitnall M, Kashem MA, Vyoral D, Richardson DR (2008). Proteomic analysis of hearts from frataxin knockout mice: marked rearrangement of energy metabolism, a response to cellular stress and altered expression of proteins involved in cell structure, motility and metabolism. Proteomics.

[86] Chu G, Kerr JP, Mitton B (2004). Proteomic analysis of hyperdynamic mouse hearts with enhanced sarcoplasmic reticulum calcium cycling. FASEB J.

[87] Labugger R, Simpson JA, Quick M (2003). Strategy for analysis of cardiac troponins in biological samples with a combination of affinity chromatography and mass spectrometry. Clin Chem.

[88] Zabrouskov V, Ge Y, Schwartz J, Walker JW (2008). Unraveling molecular complexity of phosphorylated human cardiac troponin I by top down electron capture dissociation/electron transfer dissociation mass spectrometry. Mol Cell Proteomics.

[89] Hamblin M, Friedman DB, Hill S, Caprioli RM, Smith HM, Hill MF (2007). Alterations in the diabetic myocardial proteome coupled with increased myocardial oxidative stress underlies diabetic cardiomyopathy. J Mol Cell Cardiol.

[90] Duan X, Young R, Straubinger RM (2009). A straightforward and highly efficient precipitation/on-pellet digestion procedure coupled with a long gradient nano-LC separation and Orbitrap mass spectrometry for label-free expression profiling of the swine heart mitochondrial proteome. J Proteome Res.

[91] Meng C, Jin X, Xia L (2009). Alterations of mitochondrial enzymes contribute to cardiac hypertrophy before hypertension development in spontaneously hypertensive rats. J Proteome Res.

